# Depression symptoms and cognitive function among individuals with advanced HIV infection initiating HAART in Uganda

**DOI:** 10.1186/1471-244X-10-44

**Published:** 2010-06-10

**Authors:** Noeline Nakasujja, Richard L Skolasky, Seggane Musisi, Peter Allebeck, Kevin Robertson, Allan Ronald, Elly Katabira, David B Clifford, Ned Sacktor

**Affiliations:** 1 Department of Public Health Sciences, Karolinska Institute, Stockholm, Sweden; 2 Department of Psychiatry, Makerere University, Kampala, Uganda; 3 Department of Orthopaedic Surgery, Johns Hopkins University School of Medicine, Baltimore, USA; 4 Department of Neurology, University of North Carolina, Chapel Hill, USA; 5 Department of Internal Medicine, University of Manitoba, Winnipeg, Canada; 6 Department of Medicine, Makerere University, Kampala, Uganda; 7 Department of Neurology, Washington University-St. Louis, St. Louis, USA; 8 Department of Neurology, Johns Hopkins University School of Medicine, Baltimore, USA

## Abstract

**Background:**

Among patients with HIV infection, depression is the most frequently observed psychiatric disorder. The presence of depressive symptoms and cognitive dysfunction among HIV patients has not been well studied in Sub-Saharan Africa. Initiation of highly active antiretroviral therapy (HAART) may have an effect on the prevalence and the change over time of depression symptoms and cognitive impairment among HIV-positive individuals.

**Methods:**

We recruited 102 HIV-positive individuals at risk of cognitive impairment who were initiating HAART and 25 HIV-negative individuals matched for age and education. Depression was assessed using the Centre for Epidemiologic Studies Depression Scale (CES-D). Neurocognitive assessment included the International HIV Dementia Scale (IHDS), an 8 test neuropsychological battery and the Memorial Sloan Kettering scale. Assessments were carried out at 0, 3 and 6 months.

**Results:**

The HIV-positive group had more respondents with CES-D score > 16 than the HIV-negative group at all 3 clinic visits (54%Vs 28%; 36% Vs 13%; and 30% Vs 24% respectively; all p < 0.050 OR 2.86, 95% CI: 1.03, 7.95, p = 0.044). The HIV positive group had higher likelihood for cognitive impairment (OR 8.88, 95% CI 2.64, 29.89, p < 0.001). A significant decrease in the mean scores on the CES-D (p = 0.002) and IHDS (p = 0.001) occurred more in the HIV-positive group when compared to the HIV-negative group. There was no association between clinical Memorial Sloan Kettering score and depression symptoms (p = 0.310) at baseline.

**Conclusion:**

Depression symptomatology is distinct and common among cognitively impaired HIV patients. Therefore individuals in HIV care should be screened and treated for depression.

## Background

The World Health Organisation (WHO) estimates depression to be the leading cause of disability as measured by Years Lost due to Disability (YLDs) contributing to almost 12% of all disability [1,2]. It is the second leading contributor to the global burden of disease as measured by Disability Adjusted Life Years: DALYS (the sum of years of potential life lost due to premature mortality and the years of productive life lost) in the age category of 15 - 44 years for both sexes combined [3].

HIV is a highly prevalent condition in Uganda with a current prevalence of 6% in the rural areas and 7% in the urban areas [4]. Among patients with HIV infection, clinical depression is the most frequently observed psychiatric disorder, affecting between 4% and 14% of men and women in some studies [5-7]. Indeed it has been noted that depression symptomatology rises as AIDS progresses [8]. Its rates are 2-3 times higher than those of the general community [9]. Among recently diagnosed HIV patients in South Africa the prevalence of major depression was found to be 35% [10]. Another study using the Centre for Epidemiologic Studies Depression Scale (CES-D) scale as the screening tool and the Mini International Neuropsychiatric Interview (MINI) as the diagnostic tool has shown the rate of depression to be 14% among HIV-positive individuals [11]. In Uganda, severe depressive symptomatology using a cut off of > 23 among HIV-positive individuals has been found at 47% using the CESD- depression scale as the only investigative tool [12]. In the same setting, rates for depression among first time admissions of severe mental illness have been found at 10% [13]. While the rate in patients who are comorbid with HIV and pulmonary tuberculosis has been found to be 60% [14].

Psychiatric illness is not routinely screened for in resource constrained settings and its symptoms are often not easily recognised in the health care setting [3]. Commonly there are no units for mental health service delivery thus making depression often go undetected among the HIV clientele that attend these clinics [12]. Secondly, depressed patients lose hope and motivation and may often neglect to take prescribed treatments, including antiretroviral therapy.

Cognitive impairment in HIV patients occurs on a spectrum ranging from minor cognitive motor disorder to HIV dementia, (also known as AIDS dementia complex, HIV encephalopathy and HIV associated dementia) [15]. A study designed to assess the natural history of HIV-associated affective and cognitive disorders found the prevalence of cognitive impairment and prominent depression symptomatology to be 17.9 and 15.5% respectively [16]. Individuals that are depressed may or may not show signs of cognitive dysfunction [15,17]. In some situations, depression may be the initial presentation of HIV dementia and can make cognitive impairment worse [15]. Indeed the condition is at times difficult to differentiate from HIV dementia [18]. This creates a situation in which the depression often goes undetected and untreated in many of the patients.

There is minimal epidemiological data analyzing mental health outcomes among HIV-positive and HIV-negative individuals in the African setting. We therefore undertook the following study whose aims were: (1) to assess depression symptomatology among HIV-positive patients who were about to initiate HAART and HIV-negative individuals; (2) to determine the association of depression symptomatology and cognitive function among HIV-positive and HIV-negative individuals; and (3) to evaluate changes in depression symptomatology among HIV-positive individuals receiving HAART. Our hypotheses were: (1) depression symptomatology would occur more commonly among HIV-positive individuals when compared to HIV-seronegative individuals; (2) depression symptomatology would be associated with cognitive impairment among HIV-positive individuals; and (3) following initiation of HAART, depression symptomatology would improve concomitant with improvements in immunological function.

## Methods

Ethical approval to carry out the study was received from the Makerere University Research and Ethics Committee, the Uganda National Council for Science and Technology as well as the Institutional Review Board of the Johns Hopkins University in Baltimore, Maryland. All study participants gave informed consent. Individuals who could read and write signed their names after reading through the consent form. For individuals who were unable to read, the research assistants read the form for the individual who thereafter signed with the use of a thumb print.

### Participants

A prospective cohort of HIV-positive patients at risk for cognitive impairment (as defined by a CD4 count < 200 or poor performance on a screening test for HIV dementia [19]) and HIV-negative individuals was enrolled for longitudinal assessment of depression symptomatology, cognitive function and functional status. Between September 2005 and January 2007, we recruited 102 HIV-positive individuals at the Infectious Disease Clinic, Makerere University, located at Mulago national referral hospital in Kampala, Uganda. This specialized outpatient HIV clinic provides free quality care including anti-retroviral therapy for general HIV cases. It also serves as a national referral centre, providing specialist consultations for patients who are not responding well to treatment at other health facilities. Over 10,000 HIV/AIDS clients have been registered in the clinic since its establishment in 2004. Approximately 5,600 of these clients receive life-saving antiretroviral therapy. The 25 HIV-negative age- and education-matched controls were recruited from the AIDS Information Centre (AIC), a voluntary counselling and testing centre. Selection criteria for the HIV positive individuals included: CD4 lymphocyte count < 200, ability to speak Luganda or English, clinic attendance of ≥ 2 visits in the past 6 months, residence within a 20-km radius of Kampala for the previous 6 to 12 months and unlikely to move out of the area. The exclusion criteria included age less than 18 years, an active or known past central nervous system opportunistic infection, fever of > 37.5°C, a history of a chronic neurologic disorder, active psychotic disorder, alcoholism - CAGE score of ≥2, physical deficit (e.g., amputation), a Karnofsky Performance Scale < 50 or a severe medical illness that would interfere with the ability to perform study evaluations. All above applicable criteria were used for the HIV negative group. There was no need for prior visits to the AIC in this group however they were required to have an ELISA confirmed HIV negative test.

All HIV positive subjects had a baseline CD4 lymphocyte count, viral load and were initiated on the generic co-formulated HAART regimen [Stavudine, lamivudine and Nevirapine]. Follow up was conducted at 3 and 6 months.

Details of study design have been previously published [20]. Specific elements used to answer our objectives are described below.

### Evaluation of depression

Clinical evaluation was carried out on all patients using standardized questionnaires. Depression symptomatology was assessed using the Centre for Epidemiologic Studies Depression Scale, a widely used and well-validated instrument. This 20-item short, self-reporting scale was developed by the Centre for Epidemiologic Studies and has been validated and used in the African setting [11,12,21]. It includes few vegetative symptoms, the emphasis being on the affective symptoms like depressed mood, feelings of guilt and worthlessness, psychomotor retardation, loss of appetite and sleep disorders. A score of at least 16 suggests a clinically significant level of psychological distress. In a general population, approximately 20% of individuals would be expected to score in this range. Individuals in our study with persistently high scores on the CES-D during the follow up period were referred to the psychiatric clinic for further management.

### Evaluation of cognitive impairment

Cognitive assessment included a screening test for HIV dementia using the International HIV dementia Scale (IHDS) which was validated in the same study setting previously [19]. The confirmatory cognitive tests were performed using a battery of 8 neuropsychological tests that included World Health Organization - University of California-Los Angeles Auditory Verbal Learning Test (WHO-UCLA AVLT) for verbal memory; Timed Gait and Grooved Pegboard Tests for motor performance; Symbol Digit Test and the Color Trails Test for psychomotor speed performance; Digit Span Forward and Backward for attention and working memory; Finger Tapping Test for motor performance and lastly verbal fluency was assessed with the Category Naming Test. The Karnofsky Performance tested for functional performance. A score of 10 or fewer points on the IHDS indicates that an individual has an increased likelihood of cognitive impairment associated with HIV infection. All these assessments were used to assign a Memorial Sloan Kettering (MSK) dementia stage of 0, 0.5, or ≥1 [22,23]. Details about the instruments appear in earlier publications [19,24].

### Data analysis

Comparisons were made between the HIV-positive and HIV-negative group as detailed below. All analyses were conducted using SAS statistical software package (SAS Institute, Carey, NC). Statistical significance was set at a Type I error of 0.05.

### Baseline prevalence

Baseline prevalence of depression symptomatology and cognitive impairment among HIV-positive and HIV-negative individuals was estimated as the ratio of the number of individuals meeting screening criteria on either the CES-D or the IHDS to the number of individuals completing the respective screening instrument at baseline. Chi square and Fisher's exact tests were used to compare the MSK score for cognition and the CES-D score for depressive symptoms. A 95% confidence interval (CI) for this prevalence estimate was calculated. Similarly, the proportion of HIV-positive and HIV-negative individuals with dual prevalence of depressive symptomatology and cognitive impairment was estimated.

These estimates were compared using logistic regression methods with prevalence as the dependent measure and HIV status as the independent measure to determine the likelihood of depression symptoms given HIV infection. Baseline characteristics of age and gender were included in the predictive model, as there were significant differences on these factors between the two groups.

### HAART impact on depression symptomatology

Individual participants were classified as having depression symptoms, cognitive impairment, or both at baseline and at 3- and 6-months follow-up. Differences in the likelihood of these conditions were modelled using a repeated measures logistic regression model. The model was adjusted for baseline characteristics of age, gender and HIV status. The potential for interaction between HIV status and follow-up time was tested.

## Results

The HIV-positive and HIV-negative cohorts had a comparable mean age (34.2 years versus 30.3 years) but the HIV-positive individuals were more likely to be female (73% versus 40%) and had lower baseline Karnofsky Performance Scale scores (mean 84 SD 8.5) compared to the HIV-negative individuals (mean 98 SD 4.1). The read/write language variables at baseline revealed that of the HIV-negative controls, 25/25 (100%) could read and 24/25 (96%) could write. For the HIV-positive participants, 92/103(89.3%) could read and 84/103 (81.5%) could write. These differences in reading and writing were not significant for reading (p = 0.082), but were for writing (p = 0.039) using Fisher's exact test. Baseline HIV RNA (mean, SD) for individuals with CES-D ≤ 16 was 239,720 (267,034) while for CESD of ≥ 16 it was 241,636 (240,054). The difference was not significant between the two groups (p = 0.970). The mean CD4 cell count for the HIV positive group was 130 cells/ul at baseline.

### Prevalence of depression symptoms

Based on CES-D scores, 55/102 HIV-positive individuals were identified with depression symptoms at baseline i.e. a score of ≥ 16 (Table 1). Thus, prevalence of depression symptoms was estimated to be 53.9% (95% CI 44.1%, 63.8%). Similarly, 7/25 (28%) HIV-negative individuals were identified with depression symptoms (95% CI 10.4%, 45.6%). Adjusting for differences in age and gender, the likelihood of depression symptoms was significantly higher among HIV-positive individuals compared to HIV-negative individuals (OR 2.86, 95% CI: 1.03, 7.95, p = 0.044).

**Table 1:** Demographic and Clinical Characteristics of the HIV-positive and HIV-negative individuals.

Characteristic	HIV Positive N = 102	HIV Negative N = 25	p-value^1^
Age, years (mean, SD)	34.2 (6.23)	30.3 (3.99)	.004
Female, n (%)	74 (72.6%)	10 (40.0%)	.004
Education, years (mean, SD)	9.1 (4.3)	10.4 (4.2)	.207
CD4 cell count (mean, SD)	130 (69.5)	N/A	.654
Plasma HIV RNA, log-10 copies/mL (mean, SD)	4.9 (0.87)	N/A	.672
CES-D score (mean, SD)	18.1 (11.4)	9.1 (9.0)	< .001
Karnofsky Score	84 (8.5)	98 (4.1)	0.001
**Prevalence of depression symptoms**			
Baseline (HIV+ = 102 HIV- = 25)	55 (53.9%)	7 (28.0%)	.021
3-month (HIV+ = 95 HIV- = 23)	34 (36.0%)	3 (13.0%)	.008
6-month (HIV+ = 93 HIV- = 21)	28 (30.0%)	5 (24.0%)	.206
**IHDS score (mean, SD)**	9.44 (1.8)	11.10 (0.8)	< .001
**Prevalence of cognitive impairment**			
Baseline -do-	70 (68.6%)	4 (16.0%)	< .001^2^
3-month -do-	34 (35.8%)	5 (21.7%)	
6-month -do-	28 (30.1%)	9 (42.8%)	
**Dual prevalence of depression and cognitive impairment**			
Baseline -do-	40 (39.2%)	1 (4.0%)	.013^2^
3-month -do-	34 (35.8%)	1 (4.0%)	
6-month -do-	28 (30.1%)	2 (9.5%)	

^1^Differences between HIV-positive and HIV-negative individuals were made using Student t-test (continuous measures) or Fisher's exact test (binary measures) unless otherwise noted.

^2^Differences in prevalence measures were assessed using logistic regression, adjusting for age and gender.

### Prevalence of cognitive impairment

Based on IHDS scores, 70/102 HIV-positive individuals were identified with cognitive impairment at baseline (Table 1). Prevalence of cognitive impairment was estimated to be 68.6% (95% CI 59.6%, 77.6%). Among HIV-negative individuals, 4/25 (16%) were identified with cognitive impairment (95% CI 1.6%, 30.4%). There was a greater trend for improvement on the neuropsychological battery tests among the HIV-positive subjects but the only significant change was in Color Trails 2 test (p = 0.020) after adjusting for differences in sex. There was no association between clinical MSK score and depression symptoms (p = 0.310) at baseline. After adjusting for age and gender, the likelihood of cognitive impairment was significantly higher among HIV-positive individuals compared to HIV-negative individuals (OR 8.88, 95% CI 2.64, 29.89, p < 0.001).

### Dual prevalence

Based on CES-D and IHDS scores, 40/102 HIV-positive individuals were identified with both depression symptoms and cognitive impairment (Table 1). Dual prevalence of these conditions was estimated to be 39.2% (95% CI 29.7%, 48.7%). Among HIV-negative individuals 1/25 (4%) was identified with both depression symptoms and cognitive impairment (95% CI -3.6%, 11.7%). Adjusting for age and gender, the likelihood of both depression symptoms and cognitive impairment among HIV-positive individuals was significantly higher in this group compared to HIV-negative individuals (OR 13.96, 95% CI 1.74, 111.73, p = 0.013).

### Longitudinal follow-up

The retention rate of study participants in the study was fairly good. Of the HIV-negative controls, 23/25 (92%) returned for the second visit and 21/25 (84%) returned for the third visit. Of the HIV-positive participants, 95/103(92%) returned for the second visit and 93/103 (90%) returned for the third visit.

**Figure 1:**
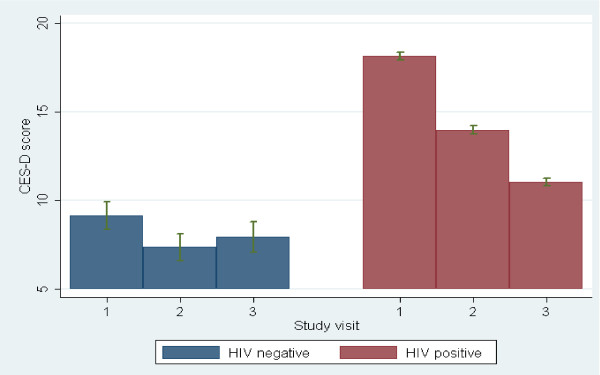
**Mean CES-D scores among HIV-negative and HIV-positive individuals**. The depressive symptoms measured with CES-D show a decline in the average score over the study period. This decline in symptoms was significant in the HIV-positive individuals p = 0.003 but not among the HIV-negative individuals p = 0.434.

Significant interaction terms indicate that HIV-positive patients experience a different long-term course in outcome compared to HIV-negative individuals. HIV-positive individuals experienced a significant decrease in the prevalence of depression symptomatology (p = 0.026), cognitive impairment (p < 0.001), and dual prevalence of these two conditions (p = 0.004) compared to HIV-negative individuals, see figure 1. At the 6 month follow up time point the difference in CES-D score was not statistically significant between the HIV positive and the HIV negative group however, the cognitive impairment was higher for the HIV-negative group. In addition to experiencing this decline in prevalence, mean scores on the CES-D and the IHDS decreased significantly among HIV-positive individuals compared to HIV-negative individuals (p = 0.003 and p < 0.001, respectively). Though the mean CD4 increased from 129 at baseline to 272 cells/ul at 6 months (p = 0.010), we noted there was no association between increasing CD4 cell count and decreasing CES-D score (p = 0.168) over the study period. Similarly, there was a marked reduction in log10 plasma HIV RNA of the HIV-positive group from 4.92 log10 copies/mL to 2.53 log10 copies/mL by the 6 months review. Using a repeated measures analysis of variance, this was a statistically significant reduction (p < 0.001).

## Discussion

We aimed to estimate the prevalence of depression symptomatology and cognitive impairment among HIV-positive individuals through a comparative study. We have shown that the presence of the depressive symptomatology and cognitive impairment was higher at baseline among HIV-positive individuals at risk of cognitive impairment compared to HIV-negative individuals. The scores generated on the cognitive tests in both groups of individuals were not biased by literacy level as the sample had a high literacy rate. Both the depression symptomatology and cognitive impairment improved after initiation of highly active antiretroviral therapy.

In the general population 20% of individuals are expected to have abnormal scores on the CES-D [21]. This instrument has been used in a number of African HIV populations and found to be valid [12,11]. The DSM-IV acknowledges that psychiatric conditions including depression can exhibit as a symptom a mild degree of cognitive impairment. On the other hand, depression can also be a major symptom of cognitive impairment and is estimated to be present in 40-50% of patients with dementia hence posing a diagnostic challenge [17,25]. Indeed, in our current study, we found a high rate of dual prevalence for both conditions.

Individuals with a high viral load have been shown to have more symptoms for depression [26]. As individuals improved immunologically with an increase in the CD4 count, there was a decrease in the symptoms of cognitive impairment and depression. However, surprising to us was the persistence of depression symptomatology well above the cut off score in a number of the study participants suggesting that the two conditions i.e. cognitive impairment and depressive symptoms can be distinct and should be treated appropriately for good treatment outcome. By the end of the follow up period, individuals in the HIV positive group had a higher prevalence of the depressive symptomatology; this may have been as a result of loss to follow up in the study group. However loss to follow up was insignificant and therefore it is unlikely that the difference between the groups is totally attributable to the difference in the attrition rates of the HIV-positive and HIV-negative groups.

Persisting depressive symptomatology can be a great contributor to disability in affected individuals[2,3]. This may have implications on the general well-being of the individual as often the depression is somatized and is usually missed in primary health care and hence is left untreated [27]. Such individuals may keep returning for clinic reviews even when they may not have done so had their depression been detected and treated early.

The HIV-positive subjects in our study were representative of the demographics of HIV infection in Uganda. The majority of HIV infected individuals seen in HIV/AIDS care are women, a group that at the same time tends to contribute more to individuals that suffer from depression as has been shown by previous studies [3,5,12]. Another study conducted by Kaharuza and other [12] showed the prevalence of depression symptomatology to be 43% which is comparable with our study findings. However in that study the cut off of 23/60 on the CES-D was considered as a case of depression. Whereas in that study the presence of a high score of symptoms was considered indicative of depression, our study used a cut off of > 16 to indicate depression symptomatology on the CES-D. The cut off of 16 for the CES-D to define depression symptomatology has been used frequently in other research studies [11,28]. Other studies found different rates ranging from 14% to 35%, probably as a result of a different methodology and the use of different diagnostic instruments [10,11].

Depression greatly affects the functioning of an individual [3]. This can be made worse by lack of detection and non-treatment, more so in individuals that may be cognitively impaired. Such a situation works against successful treatment of HIV infected patients, making it imperative to diagnose and effectively treat depression to improve the outcome of antiretroviral therapy. In this study the symptomatology for depression kept on decreasing with time with an inverse increase in the functionality of the individual. Depression as a mood disorder can be a self limiting condition. However given the burden of disease the condition brings about, it should be treated whenever it is detected in an individual.

The observed decrease in the CES-D scores may be a result of the improving health of the individuals brought about by antiretroviral therapy. However, even though the CD4 count increased in these study patients they also had a persistently high average CES-D score over the study period. This finding emphasizes the fact that depression symptomatology does not fully resolve with antiretroviral therapy. Therefore, appropriate treatment with antidepressant therapy should be instituted for such patients. Whereas the CES-D-scale is only a screening instrument that may pick up symptoms of depression, the individuals that remained with depressive symptomatology (30%) could possibly be diagnosed as having a major depression disorder were they to be subjected to DSM-IV research diagnostic criteria. Indeed such patients require antidepressant medication that, unfortunately they were not receiving.

This study was not without limitations. The comparative group included only 25 HIV-negative individuals for statistical and practical reasons. Practically it was more difficult to recruit the HIV-negative individuals and retain them in the follow-up. They had no reason to return as they had no ailment or need for medication. Secondly, because these individuals are neurologically normal, they, as a group, exhibit less variability on the outcome measures. Therefore, we needed to enroll and follow a smaller number. However the sample recruited in both the HIV-positive and HIV-negative groups was sufficient to show statistical difference in the outcome measures.

## Conclusions

Depression symptomatology is distinct and common among cognitively impaired HIV patients. A large number of individuals that are HIV infected in Sub-Saharan Africa, do not receive care. It is important therefore, that those who attend HIV care are screened for depression symptoms and cognitive dysfunction. This would make it possible to manage both conditions appropriately so as to improve patient outcomes.

## Competing interests

The authors declare that they have no competing interests.

## Authors' contributions

NN participated in the design and coordination of the of the study, RLS participated in the design of the study and performed the statistical analysis, SM participated in the conception of the study, PA helped in the drafting of the manuscript, KR participated in the design and interpretation of study findings, AR participated in the design and drafting of the manuscript, EK participated in the coordination of the study, DBC participated in the monitoring of study evaluations and NS conceived the study, participated in its design, coordination as well as drafting of the manuscript. All authors read and approved the final manuscript.

## Pre-publication history

The pre-publication history for this paper can be accessed here:

http://www.biomedcentral.com/1471-244X/10/44/prepub
